# Stage-dependent survival in gastric cancer: a Danish nationwide cohort study

**DOI:** 10.1007/s00423-025-03861-y

**Published:** 2025-10-23

**Authors:** Oliver Nørholm Kempf, Lise Bech Jellesmark Thorsen, Nikolaj Nerup, Daniel W. Kjær, Jonas Sanberg, Mette Siemsen, Sarunas Dikinis, Michael Stenger, Rajendra Singh Garbyal, Lene Bæksgaard, Michael Patrick Achiam

**Affiliations:** 1 Department of Transplantation and Digestive Diseases, Copenhagen University Hospital Rigshospitalet, Inge Lehmanns Vej 7, 2100 Copenhagen, Denmark; 2 Department of Oncology, Aarhus University Hospital, Aarhus, Denmark; 3 Department of Surgery, Aarhus University Hospital, Aarhus, Denmark; 4 Department of Surgery, Odense University Hospital, Odense, Denmark; 5 Department of Cardiothoracic Surgery, Copenhagen University Hospital Rigshospitalet, Copenhagen, Denmark; 6 Department of Gastrointestinal Surgery, Aalborg University Hospital, Aalborg, Denmark; 7 Department of Cardiothoracic and Vascular Surgery, Odense University Hospital, Odense, Denmark; 8 Department of Pathology, Copenhagen University Hospital Rigshospitalet, Copenhagen, Denmark; 9 Department of Oncology, Copenhagen University Hospital Rigshospitalet, Copenhagen, Denmark; 10 Department of Clinical Medicine, University of Copenhagen, Copenhagen, Denmark

**Keywords:** Gastric cancer survival, Chemotherapy, Surgery, Cancer stage

## Abstract

**Background:**

Gastric cancer remains a major clinical challenge with poor prognosis. This study investigated survival outcomes based on treatment strategy, tumor stage, and histology in Danish gastric cancer patients.

**Methods:**

From January 2013 to December 2021, 2,156 gastric cancers were registered in the Danish Esophagogastric Cancer Group database, covering 99% of national cases. Data were analyzed for patients with intestinal and diffuse-type cancers. Survival was assessed using Kaplan–Meier curves and Cox regression, adjusting for tumor stage, treatment, and demographics.

**Results:**

Median survival was significantly higher with surgery ± perioperative chemotherapy (SCT) than with palliative treatment. For the intestinal-type cancers, SCT resulted in a median survival of 45.2 months (95% CI [35.4–55.1]) versus 5.1 months (95% CI [4.6–5.7]) with palliative treatment. Patients with diffuse type, treated with SCT had a median survival exceeding 128 months, compared with 6.3 months (95% CI [5.2–7.5]) with palliative treatment. Patients receiving epirubicin based CT had a lower risk of death (HR 0.74, *p* = 0.04) compared with upfront surgery, while FLOT (5-fluorouracil, leucovorin, oxaliplatin, and docetaxel) similarly reduced the risk of death (HR 0.69, *p* = 0.04). No significant difference was observed between the two CT regimens. Palliative CT and radiotherapy improved survival over best supportive care (*p* < 0.001). Advanced tumor stage was associated with worse survival, while the histological subtype had no impact on overall survival outcomes.

**Conclusion:**

This study emphasizes the survival benefit of multimodal treatment strategies, especially surgery combined with perioperative CT. Palliative interventions also improved outcomes in advanced disease.

**Supplementary Information:**

The online version contains supplementary material available at 10.1007/s00423-025-03861-y.

## Introduction

Gastric cancer is the fifth most prevalent cancer globally and the fourth leading cause of cancer-related mortality [1]. In Denmark, approximately 250 persons are diagnosed with gastric cancer each year [2]. The most common curative intended treatment for gastric adenocarcinoma is surgical resection, while palliative CT is advised for unresectable tumors or recurrent disease [3].

According to the Union for International Cancer Control (UICC), gastric cancers can be divided into cardia cancers and non-cardia cancers. The UICC 8th edition defines tumors involving the esophagogastric junction (EGJ) within the proximal 2 cm of the cardia as esophageal cancers. Tumors located 2 cm distal from EGJ should be staged as gastric cancers [4]. Risk factors for gastric cancer vary according to the anatomical subsite of the disease. Non-Cardia gastric cancers account for 80 percent of all gastric cancers globally and have been associated with *Helicobacter pylori* infection, high alcohol, salt intake, and poor diet [5].

Although the staging systems have been widely used for the stratification of patients and prediction of prognosis, discrepancies in survival among patients within the same stage are frequently observed [6]. Prognosis varies among individuals and is influenced by factors such as tumor stage, lymph node involvement, the presence of a microscopic positive resection margin, response to perioperative CT, age, sex, and pathological subtype [7–10].

Selecting the appropriate treatment for gastric cancers poses a challenge. While surgery and oncological treatments can extend survival, they also come with significant risks of complications [11, 12]. This study utilized the Danish Esophago-Gastric Cancer (DEGC) group's national database, offering comprehensive follow-up data for all biopsy-verified cancers. Remarkably, the DEGC database has maintained an exceptional 99% coverage rate since 2012. The study's robustness is further strengthened by its integration with the Danish Civil Person Registry (CPR), which systematically records all healthcare data in Danish residents from birth to death [13].

The aim of this study was to map survival amongst Danish patients diagnosed with gastric cancer. Furthermore, we intend to create a simple schematic survival overview to assist surgeons, oncologists, and patients in making informed treatment choices. This was based on a pragmatic, intention-to-treat analysis of national registry data.

## Methods

### Patients

This study adhered to the STROBE checklist for cohort studies [14]. All patients diagnosed with gastric cancer between the 1^st^ of January 2013 and the 31^st^ of December 2021 were included. Patients were registered in the DEGC database, which consisted of patients from the four upper GI centers in Denmark: Copenhagen University Hospital Rigshospitalet, Odense University Hospital, Aarhus University Hospital, and Aalborg University Hospital. The database included detailed records of all surgical and oncological procedures, as well as information on comorbidities and survival for all biopsy-confirmed gastric carcinomas linked to CPR numbers. While some data were manually entered, others were automatically retrieved through the Danish CPR system. Patients were stratified according to their treatment allocation. Treatment allocation consisted of *Surgery* ± CT (SCT) and *Palliative* treatment. *SCT* patients received perioperative CT before surgery or upfront surgery. Surgery consisted of endoscopic resection for superficial tumors (T1N0M0) and total or subtotal gastrectomy for higher stages [15]. Palliative treatment consists of best supportive care, chemo(radio)therapy, and, in some instances, palliative surgery. Radiation therapy for palliative patients consisted of a dose of 30 GY or less. Palliative surgery consisted of endoscopically placed stents, bypass procedures, or resectional surgery (resection with a gastrojejunal anastomosis or gastrectomy with a Roux anastomosis) [16]. 

If patients had more than one treatment protocol linked to their social security number, only the first treatment intention was selected.

### Clinicopathological factors

The UICC 8^th^ edition TNM classification for gastric cancers was used to generate the clinical stage and pathological stage [4]. Pathological TNM staging was available in patients who received SCT (ypTNM) and in those who received up-front surgery (pTNM).

The histopathological classification of the tumors is based on a combination of the Lauren classification and the WHO's 5th edition (2019) classification of digestive system tumors [17]. According to Lauren, tumors are broadly classified into two categories: diffuse and intestinal. The WHO classification enhances this categorization. The diffuse type includes poorly cohesive and signet ring cell phenotype. The intestinal type comprises adenocarcinomas, including tubular, papillary, and poorly differentiated forms. Mucinous adenocarcinomas are categorized separately as a third group. Other histopathological subtypes, such as squamous cell carcinoma, undifferentiated carcinoma, adenosquamous carcinomas, and metastases, are collectively grouped and categorized as'others'. Neuroendocrine and gastrointestinal stromal tumors were not included in the database. Survival analyses were conducted on the intestinal and diffuse type groups. A detailed description of the histopathological distribution is available in Supplementary Table 1.

Due to missing data, approximately 95% of patients were successfully staged. Staging was incomplete when the primary tumor (T) or regional lymph node status (N) was missing or not accessible. Further statistical analyses were conducted only on staged patients with available histopathology.

### Endpoint

The primary endpoint was all-cause mortality, measured from the date of diagnosis. Follow-up was censored at the earliest of the following events: date of death, date of emigration, or end of study follow-up (September 12, 2023). Patients alive at the end of follow-up were censored on this date.

### Statistical analysis

This study used Kaplan–Meier methods to estimate survival curves, median survival, and cumulative survival rates, with 95% confidence intervals (CI) calculated using Greenwood’s formula. The Mantel-Cox (log-rank) and Wilcoxon tests compared survival differences across stages. Median follow up was calculated using reverse the Kaplan–Meier technique. A multivariate analysis using Cox proportional hazards regression controlled for confounders, including TNM stage, American Society of Anesthesiologists (ASA) score, Charlson Comorbidity Index (CCI), age, and treatment type. For resected patients, we analyzed the impact of pathological stage, perioperative CT vs. upfront surgery. For palliative patients, we assessed the effects of CT and radiotherapy. Hazard ratios (HR) for death are reported with 95% CIs and survival beyond the 128-month observation period was noted as not reached (NR). For certain subgroups, such as diffuse-type cancers treated with surgery ± perioperative chemotherapy, the median overall survival exceeded the maximum observation time of 128 months. These estimates should therefore be interpreted with caution until longer-term follow-up data become available. A p-value < 0.05 indicated statistical significance. Statistical analyses were conducted using IBM SPSS statistics (version 29–0-1–0 (171) and R studio (version 2024.04.2 + 764) software. In this study, AI was utilized to generate code for R and SPSS and to correct grammatical errors.

## Results

This study included 2,156 patients diagnosed with gastric cancer between January 1, 2013, and December 31, 2021. Among palliative patients, the median follow-up time was 85 months (95% CI: 55–114), with 25 patients censored. For patients undergoing surgery, the median follow-up time was 72 months (95% CI: 67–79), with 324 patients censored. The median age was 72 years (IQR = 62–80), with a male-to-female ratio of 1.33 (1,229 males and 927 females). Most patients were treated with palliative intent (*n* = 1,438; 66%), while the remaining 718 patients (33%) underwent SCT. Intestinal-type tumors accounted for 79% (*n* = 1,710) of cases, while the diffuse types comprised 11% (*n* = 244). Staging was achieved in 95% of the intestinal types and 94% of the diffuse types. All patients undergoing surgery were pathologically staged. A detailed description of patient demographics is found in Table 1.

**Table 1:** Gastric cancer demographics

Variable				No. Patients	(%)
Age (median): 72 years (IQR = 62–80)				2156	
Sex					
	Male			1229	(57)
	Female			927	(43)
ASA Score					
	1			181	(8)
	2			872	(40)
	3			689	(32)
	4			279	(13)
	5			40	(2)
	Missing			95	(5)
Charlson Comorbidity Index					
	0			994	(46)
	1			422	(20)
	2			346	(16)
	3			158	(7)
	≥ 4			236	(11)
Treatment status				2156	
	Palliative			1438	(66)
		Chemotherapy			
			Yes	597	
			No	841	
		Radiotherapy		139	
	Surgery ± perioperative chemotherapy			718	(33)
		EOX/ECF		303	
		FLOT		151	
		Upfront surgery		264	
Histology					
	Intestinal type			1710	(79)
	Diffuse type			244	(11)
	Others incl. Mucinous adenocarcinomas			126	(6)
	Missing			76	(4)
Pathological stage				660	
	Diffuse type			95	(14)
	Intestinal type			565	(86)

Table 1. illustrates the demographics of gastric cancers included in this study

*ASA* American Society of Anesthesiologists

*FLOT* 5-fluorouracil, leucovorin, oxaliplatin, and docetaxel

*EOX* Epirubicin, oxaliplatin, and capecitabine

*ECF* Epirubicin, cisplatin, and 5-fluorouracil

### Intestinal type survival

**Fig. 1:**
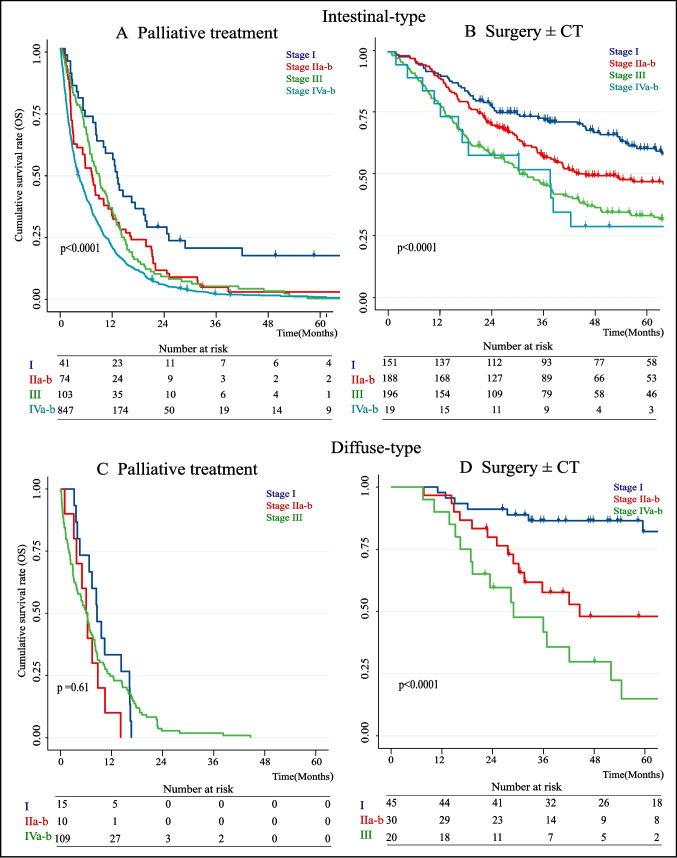
Shows Kaplan Meier survival curves for the gastric cancer patients stratified by UICC stage, Lauren classification and treatment intention. Survival curves are visualized as cumulative survival rates as a function of the time (Months). Each curve is accompanied by a number at risk table

Patients treated with palliative intent had a median overall survival (mOS) of 5.1 months (CI 95% [4.6–5.7]). A significant difference in survival over time was observed between stages (*p* < 0.001). Stage I had the best mOS of 12.8 months (95% CI [11.0–14.7]), while Stage IVa-b had the poorest mOS of 4.1 months (95% CI [3.6–4.7]). At five years follow-up, only 2% (95% CI [1-3]) of patients were alive. (Table 2, Fig. 1A).

**Table 2:** Gastric cancer survival

**Intestinal type**						
**Treatment**	**Stage**	***N***	**Median survival**	**Survival rates**		
			(95% CI)	1 year	3 years	5 years
Palliative						
	I	41	12.8 (11.0–14.7)	0.56 (0.43–0.74)	0.21 (0.11–0.39)	0.18 (0.09–0.36)
	IIab	74	7.1 (4.7–95)	0.32 (0.23–0.45)	0.07 (0.03–0.16)	0.04 (0.01–0.13)
	III	103	8.8 (7.0–10.6)	0.34 (0.26–0.44)	0.06 (0.03–0.13)	0.01 (0.001–0.07)
	IVab	847	4.1 (3.6–4.7)	0.21 (0.18–0.24)	0.03 (0.02–0.04)	0.01 (0.008–0.03)
	Overall	1065	5.1 (4.6–5.7)	0.24 (0.22–0.27)	0.04 (0.03–0.05)	0.02 (0.01–0.03)
Surgery ± CT						
	I	151	106.6 (63.4–150)	0.91 (0.86–0.96)	0.72 (0.65–0.80)	0.61 (0.53–0.70)
	IIab	188	45.2 (25.3–65.2)	0.89 (0.85–0.94)	0.57 (0.50–0.65)	0.47 (0.40–0.56)
	III	196	31.9 (24.9–38.9)	0.79 (0.73–0.85)	0.46 (0.39–0.53)	0.33 (0.27–0.41)
	IVab	19	37.2 (12.4–61.9)	0.79 (0.63–0.99)	0.52 (0.34–0.81)	0.29 (0.14–0.60)
	Overall	554	45.2 (35.4–55.1)	0.85 (0.82–0.88)	0.57 (0.53–0.61)	0.45 (0.41–0.50)
**Diffuse type**						
**Treatment**	**Stage**	**N**	**Median survival**	**Survival rates**		
			(95% CI)	1 year	3 years	5 years
Palliative						
	I	15	8.6 (5.8–11.4)	0.33 (0.16–0.68)	0 (NA)	0 (NA)
	IIab	10	6.0 (4.1–8.0)	0.10 (0.02–0.64)	0 (NA)	0 (NA)
	IVab	109	6.3 (4.5–8.0)	0.25 (0.18–0.34)	0.02 (0.01–0.07)	0 (NA)
	Overall	134	6.3 (5.2–7.5)	0.25 (0.18–0.33)	0.01 (0.01–0.05)	0 (NA)
Surgery ± CT						
	I	45	NR	0.98 (0.94–1)	0.86 (0.77–0.97)	0.82 (0.70–0.96)
	IIab	30	44.6 (NA)	0.96 (0.91–1)	0.58 (0.42–0.79)	0.48 (0.32–0.72)
	III	20	28.9 (12.9–45.0)	0.90 (0.77–1)	0.42 (0.24–0.72)	0.15 (0.01–0.47)
	Overall	95	NR	0.96 (0.92–0.99)	0.68 (0.59–0.79)	0.57 (0.47–0.69)

Table 2. Median survival and cumulative 1-, 3-, and 5-year survival rates in intestinal-type adenocarcinomas and diffuse-type adenocarcinomas, stratified by the UICC 8th edition staging system and treatment strategy: Survival data are presented for patients receiving palliative treatment and those undergoing surgery with or without perioperative chemoradiotherapy (CT), reflecting clinical stages. All cumulative survival rates are reported as decimals and accompanied by their 95% confidence intervals. Median survival estimates were presented in months calculated using the Kaplan–Meier method. If the median survival exceeds the study’s observational period of 128 months, its denoted as not reached (NR)]. Not available [NA]

For patients undergoing surgery, the mOS was 45.2 months (95% CI [35.4–55.1]). A significant difference in survival over time was observed between stages (*p* < 0.001). Stage I had the highest mOS of 106.6 months (95% CI [63.4–150.0]), while Stage III had the lowest of 31.9 months (95% CI [23.4–37.1]). After five years, 45% (95% CI [41–49]) of patients who underwent surgery were alive. Increasing stages were associated with worse survival over time (*p* < 0.001) (Table 2, Fig. 1B).

### Diffuse type survival

For palliative patients, no significant difference in survival over time was observed across all stages (*p* = 0.61). These patients had an mOS of 6.3 months (95% CI [5.2–7.5]). Stage I patients had a median survival of 8.6 months (95% CI [5.8–11.4]), while Stage IVa-b patients had a median survival of 6.3 months (95% CI [4.5–8.0]). At the three-year mark, no survivors remained in Stages I–III, while Stage IVa-b had an OS of 2% (95% CI [1-7]). No patients survived beyond five years. (Table 2, Fig. 1C).

Among patients undergoing surgery, a significant difference in survival across stages over time was observed (*p* < 0.001); however, the mOS exceeded the observation time of 128 months (95% CI [NA]). After five years, 57% (95% CI [47–67]) of patients were alive. (Table 2, Fig. 1D).

### Univariate analysis

We analyzed the prognostic survival factors presented in Table 3 to identify variables that were independently associated with survival. Among all patients, an increase in the clinical stage was associated with a higher mortality compared with Stage I. Diffuse type was linked to a lower HR for death (0.79, 95% CI [0.67–0.92]) compared with intestinal type. Additionally, increasing age, ASA score, and CCI score were significantly associated with higher HR for death.

**Table 3:** Univariate survival analysis

Variable			HR (95%CI)	*P*
Age (years)				
	0–50		Ref	** < *0.001***
	50–60		1.09 (0.86–1.37)	*0.50*
	60–70		1.20 (0.96–1.48)	*0.11*
	70–80		1.58 (1.28–1.94)	** < *0.001***
	> 80		2.25 (1.88–2.83)	** < *0.001***
Histology				
Intestinal vs Diffuse			0.79 (0.67 - 0.92)	***0.002***
Clinical stage				
		I	Ref	** < *0.001***
		IIab	1.77 (1.40 - 2.23)	** < *0.001***
		III	2.49 (2.00 - 3.11)	** < *0.001***
		IVab	7.66 (6.26- 9.38)	** < *0.001***
ASA score				
		1	Ref	** < *0.001***
		2	1.43 (1.17 - 1.76)	** < *0.001***
		3	2.58 (2.10–3.17)	** < *0.001***
		≥ 4	5.49 (4.39 - 6.87)	** < *0.001***
Charlson comorbidity score				
		0	Ref	** < *0.001***
		1	1.04 (0.90 - 1.18)	*0.62*
		2	1.38 (1.21 −1.59)	** < *0.001***
		3	1.22 (1.01 - 1.48)	***0.040***
		≥ 4	1.98 (1.69- 2.32)	** < *0.001***
Perioperative treatment*				
	No chemo		Ref	
	EOX/ECX		0.80 (0.55–1.00)	***0.05***
	FLOT		0.74 (0.55–0.99)	***0.04***
	FLOT vs. EOX/ECX		1.08 (0.81–1.45)	***0.60***
Pathological stage*				
		0	Ref	** < *0.001***
		Iab	2.40 (0.86 - 6.71)	***0.01***
		IIab	5.49 (2.02 - 14.91)	** < *0.001***
		IIIac	14.04 (5.49 - 37.89)	** < *0.001***
		IV	8.55 (2.41 - 30.31)	** < *0.001***
Chemotherapy y/n ┼			0.54 (0.38 - 0.78)	** < *0.001***
Radiotherapy y/n ┼			0.83 (0.69 - 1.00)	*0.055*

Table 2: Independent prognostic survival factors for gastric cancer patients. Calculations are estimated using Cox proportional hazards regression. All hazard rates for death (HR) are presented as decimals and accompanied by their 95% confidence intervals. A p-value of < 0.05 indicates statistical significance. Binary comparisons are presented with the reference treatment strategy last. Patients treated before 2017 serves as reference for implementation FLOT

[*] Patients undergoing Surgery ± nCRT patients

[**┼**] Patients receiving palliative treatment

*ASA* American Society of Anesthesiologists

*FLOT* 5-fluorouracil, leucovorin, oxaliplatin, and docetaxel

*EOX* Epirubicin, oxaliplatin, and capecitabine

*ECF* Epirubicin, cisplatin, and 5-fluorouracil

For patients receiving palliative treatment, CT (HR = 0.54, 95% CI [0.38–0.78], *p* < 0.001) was significantly associated with improved survival compared with best supportive care. Radiotherapy (HR = 0.83, 95% CI [0.69–1.00], *p* = 0.055) also showed a trend toward improved survival, though it did not reach statistical significance (Table 3).

For surgically treated patients, those receiving perioperative CT had improved survival compared with those undergoing upfront surgery. The use of epirubicin-based protocols (EOX/ECX) was associated with a significantly reduced hazard of death (HR 0.80, 95% CI [0.55–1.00], *p* = 0.05), while patients treated with 5-fluorouracil, leucovorin, oxaliplatin, and docetaxel (FLOT) also showed a survival benefit (HR 0.74, 95% CI [0.55–0.99], *p* = 0.04). No significant difference was observed between FLOT and EOX/ECX (*p* = 0.60). For pathological staging, each increase in stage was consistently associated with a higher HR for death compared with Stage 0.

### Multivariate analysis

In the multivariate analysis, patients were divided into two groups: those receiving palliative treatment and those undergoing SCT. Among palliative patients, increasing age was associated with a lower HR (0.71, 95% CI [0.53–0.94], *p* = 0.01) for those aged 80 +, while no significant survival differences were observed for younger age groups. In contrast, for surgical patients, increasing age was linked to higher mortality risk, with HR 1.78 (95% CI [1.13–2.82], *p* = 0.02) for patients aged 70–80 and HR 2.00 (95% CI [1.17–3.41], *p* = 0.01) for those aged 80 +. Histopathological type was not significantly associated with survival in either group.

For clinical staging, higher stages were consistently linked to increased HR for death across both groups. Among palliative patients, higher stages were consistently associated with worse survival. In the surgical group, Stage III (HR 1.72, 95% CI [1.24–2.39], *p* = 0.001) and Stage IV (HR 2.44, 95% CI [1.31–4.58], *p* = 0.01) showed significantly decreased survival, whereas no significant difference was found between Stage I and Stage II (*p* = 0.07).

An ASA score of 1–3 had no significant effect on survival among surgical patients; ASA score 4 showed an increased HR of 4.56 (CI 95% [1.86–11.17], *p* = 0.001). In the palliative group, an increase in ASA score was significantly associated with decreased survival. CCI scores of 1–3 had no significant impact on HR in the palliative group. In the surgical group, a CCI of 3 was associated with an increased HR of 1.35 (95% CI [1.01–1.80], *p* = 0.04). However, a CCI ≥ 4 was significantly associated with decreased survival in both groups.

Among palliative patients, CT remained significantly associated with a lower HR for death of 0.36 (95% CI [0.31–0.41], *p* < 0.001). Similarly, radiotherapy continued to show a significant association with a reduced HR of 0.79 (95% CI [0.65–0.97], *p* = 0.002). (Table 4).

**Table 4:** Multivariate survival analysis

Surgery ± nCT					Palliative treatment		
Variable			HR (95%CI)	*P*	Variable	HR (95%CI)	*P*
Age (years)							
	0–50		Ref	***0.003***	0–50	Ref	*0.15*
	50–60		1.05 (0.64—1.73)	*0.84*	50–60	0.87 (0.65—1.16)	*0.33*
	60–70		1.16 (0.73—1.84)	*0.53*	60–70	0.83 (0.63—1.08)	*0.17*
	70–80		1.78 (1.13—2.81)	***0.01***	70–80	0.79 (0.61—1.03)	***0.08***
	80 +		2.00 (1.17—3.41)	***0.01***	80 +	0.71 (0.53—0. 94)	***0.02***
Histology ┼			0.92 (0.65—1.31)	*0.64*		1.17 (0.96—1.41)	*0.11*
Clinical stage							
		I	Ref	***0.01***	I	Ref	** < *0.001***
		IIab	1.35 (0.97—1.87)	*0.07*	IIab	2.59 (1.75–3.82)	** < *0.001***
		III	1.72 (1.24—2.39)	***0.001***	III	2.45 (1.69—3.55)	** < *0.001***
		IVab	2.44 (1.31—4.58)	***0.01***	IVab	4.30 (3.08—6.02)	** < *0.001***
ASA score							
		1	Ref	***0.001***	1	Ref	***0.01***
		2	1.03 (0.73—1.45)	*0.87*	2	1.24 (0.94—1.63)	*0.13*
		3	1.32 (0.90—1.94)	*0.15*	3	1.38 (1.04—1.83)	***0.03***
		4	4.56(1.86—11.17)	** < *0.001***	≥ 4	1.56 (1.15—2.11)	***0.004***
Charlson comorbidity score							
		0	Ref	*0.07*	0	Ref	***0.01***
		1	1.05 (0.77—1.43)	*0.78*	1	0.94 (0.80—1.11)	*0.47*
		2	1.35 (1.01 −1.80)	***0.04***	2	0.99 (0.83—1.18)	*0.87*
		3	1.39 (0.90—2.15)	*0.13*	3	0.90 (0.71—1.15)	*0.41*
		≥ 4	1.62 (1.07—2.45)	***0.02***	≥ 4	1.34 (1.10—1.63)	***0.003***
Chemotherapy							
	Upfront surgery		Ref	*0.08*	**Yes/no**	0.36 (0.31—0.42)	** < *0.001***
	EOX/ECX		0.74 (0.55—0.99)	***0.04***			
	FLOT		0.69 (0.48—0.99)	***0.04***	Radiotherapy*	0.79 (0.65—0.97)	***0.03***
	FLOT vs. EOX/ECX		1.08 (0.79—1.47)	*0.64*			
Pathological stage							
		0	Ref	** < *0.001***			
		Iab	2.08 (0.73—5.88)	*0.17*			
		IIab	3.99 (1.46—10.88)	***0.01***			
		IIIac	10.00 (3.67—27.23)	** < *0.001***			
		IV	8.67 (2.42—31.11)	** < *0.001***			

Table 3: Adjusted hazard rates for death (HR) for gastric cancer patients. Calculations are estimated using Cox proportional hazards regression. All HR are presented as decimals and accompanied by their 95% confidence intervals. A *p*-value of < 0.05 indicates statistical significance. The model is stratified by treatment intention, and therefore some variables are unique to one each stratification. Binary categorical comparisons are presented with the reference last

*No radiotherapy serves as reference

**┼** Intestinal type serves as reference

*ASA* American Society of Anesthesiologists

*FLOT* 5-fluorouracil, leucovorin, oxaliplatin, and docetaxel

*EOX* Epirubicin, oxaliplatin, and capecitabine

*ECF* Epirubicin, cisplatin, and 5-fluorouracil

Patients receiving perioperative CT before surgery demonstrated a reduced HR for death compared with those undergoing upfront surgery. FLOT treatment significantly reduced the HR for death to 0.70 (95% CI [0.48–0.99], *p* = 0.04). EOX/ECX treatment also showed improved survival with an HR of 0.73 (95% CI [0.55–0.98], *p* = 0.03). No significant difference in survival was observed between FLOT and EOX/ECX treatments (*p* = 0.71). Increased pathological stage was still associated with an increased HR for death, although no significant difference was observed between Stage 0 and Stage Iab (*p* = 0.17). For the remaining stages, increasing stage was linked to a higher HR for death compared with Stage 0: Stage IIab HR 3.99 (95% CI [1.46–10.86], *p* = 0.01), Stage IIIac HR 10.34 (95% CI [3.69–27.32], *p* < 0.001), and Stage IV HR 9.99 (95% CI [2.90–34.55], *p* < 0.001).

## Discussion

This study represents one of the most comprehensive reports to date on survival outcomes following treatment for gastric cancer. Overall, higher cancer stages were associated decreased survival, with this effect being even more pronounced in pathological stages. No significant difference in survival was observed between the intestinal and diffuse-type tumors. Among surgical patients, those who received perioperative CT demonstrated improved survival outcomes. For palliative patients, those receiving CT or radiotherapy had increased survival compared with those receiving only the best supportive care.

In this study, surgical patients benefitted significantly from perioperative CT in addition to surgery (EOX/ECX: HR 0.74, 95% CI [0.55–0.98], *p* = 0.04, FLOT: HR 0.69, 95% CI [0.48–0.99], *p* = 0.04). This is in line with multiple randomized trials demonstrating improved survival outcomes with perioperative treatment compared with surgery alone. The MAGIC trial reported increased 5-year survival rates and higher R0 resection rates with 75% of the tumors classified as gastric cancers [18]. These findings were supported by the FNCLCC/FFCD study, which reported similar survival benefits with 25% of the study population having gastric cancer [19]. The FLOT4 study, referenced in the most recent guidelines for esophageal and gastric cancer treatment, demonstrated survival benefits with perioperative FLOT compared with ECF/ECX [20]. However, we did not observe a significant difference in survival between patients treated with FLOT and those treated with EOX/ECX. During the study period, perioperative regimens in Denmark transitioned from epirubicin-based (EOX/ECX/ECF) protocols as supported by the MAGIC trial [18] to the FLOT regimen implemented in Denmark in 2017 [20]. While FLOT has been associated with improved survival in clinical trials, these findings suggest that in a real-world setting, the transition from EOX/ECX to FLOT may not have provided an additional survival benefit beyond what was already achieved with epirubicin-based regimens. This aligns with another Danish study on the implementation of FLOT4 in esophagogastric carcinomas, which also found no significant survival benefit compared with EOX/ECX. However, lower tolerability of FLOT4 led to a reduced completion rate, potentially explaining the lack of survival benefits [21]. Similarly, this may be a factor in the present study, although tolerability could not be assessed.

It is important to note that, as this is a national registry study, the implementation of FLOT was not uniform across all centers, warranting caution when interpreting its direct impact on survival outcomes. Additionally, the observed survival improvements over time may also reflect advancements in surgical techniques, such as minimally invasive techniques, and enhanced preoperative staging.

Chemo- and radiotherapy increased the survival amongst palliative patients. An extensive Cochrane review consisting of 60 randomized controlled trials, including 11,698 palliative patients receiving CT for gastric cancer, found that palliative CT increased survival by seven months compared with the best supportive care [22]. Based on these findings, the Danish guidelines recommend either two-drug or three-drug CT for patients, depending on treatment goals, performance status, side effect profile, and patient preferences [23]. Recommendations now also include HER2-directed therapy and immunotherapy for selected subgroups [24, 25]. Few clinical trials support the effect of palliative radiotherapy. The relatively small number of patients in this study receiving external beam radiation received a dose ranging from 20 to 35 Gy. The benefits of radiotherapy are mainly based on clinical experience, showing relief of symptoms such as malign obstruction, bleeding, and dysphagia. A randomized controlled trial (TROG 03.01) comparing palliative radiotherapy alone with chemoradiotherapy in advanced esophageal cancer found no significant differences in dysphagia relief or progression-free survival between the two approaches. However, chemoradiotherapy was associated with significantly higher toxicity rates, reinforcing that radiotherapy alone remains a viable and less burdensome option for palliation [26]. Furthermore, a systematic review demonstrated that palliative radiotherapy for gastric cancer showed symptom relief rates of 74% for bleeding, 67% for pain, and 68% for obstruction [27]. This supports the notion that CT and radiotherapy are effective for symptomatic relief and increase survival in advanced stages of disease. Selection bias is an inherent limitation in this study, as treatment allocation is not randomized but influenced by factors such as patient performance status, comorbidities, and tumor characteristics. Consequently, differences in survival outcomes may partly reflect underlying patient characteristics rather than the direct effect of CT or radiotherapy.

We found no difference in survival between intestinal type- and diffuse-type tumors after adjusting for confounding covariates. A large American study observed a similar lack of survival difference between the two when adjusted for stage [28]. A French study also reported no survival differences in early gastric cancer [29]. These findings suggest that diffuse-type cancers may have a worse prognosis in advanced stages compared with intestinal types, which is supported by a Japanese study identifying diffuse-type as a poor prognostic factor in advanced gastric cancer [30]. Interestingly, the diffuse-type was associated with a lower risk of death as an independent survival predictor, which may be related to the younger age of diffuse-type patients and their generally less advanced cancer. The high survival rates among these patients may be attributed to prophylactic gastrectomy. In this study, a subset of the diffuse-type patients underwent gastrectomy even at early stages, which could have contributed to their significantly improved survival outcomes. This aligns with clinical guidelines recommending prophylactic gastrectomy for individuals with genetic predispositions, such as pathogenic variants in the CDH1 gene, associated with hereditary diffuse gastric cancer [31]. These procedures aim to lower cancer risk and enhance long-term survival by preventing the onset of malignancy in high-risk individuals. Considering the strong hereditary component in diffuse-type tumors, early surgical intervention may have been guided by family history or genetic analysis. The absence of observed survival differences in the adjusted analysis may also point to the effectiveness of current multimodal therapies in managing the aggressive nature of diffuse-type cancers. However, this study included prophylactic gastrectomy’s only when a tumor was detected through pathological assessment of the resected specimen, meaning it does not fully capture the true value of prophylactic gastrectomy in patients without detectable tumors. Additionally, the relatively small cohort limits statistical power for these comparisons and warrants cautious interpretation.

Lastly, we found that an increase in the pathological stage was associated with a significantly higher risk of death compared with a corresponding increase in the clinical stage. This difference likely reflects the impact of neoadjuvant treatment on staging and outcomes. Patients who responded well to neoadjuvant therapy were downstaged, migrating to a lower pathological stage, which often corresponded to better survival outcomes. In contrast, non-responders either remained in their initial stage or experienced disease progression, resulting in higher pathological stages and decreased survival. These findings highlight the prognostic value of pathological staging in capturing treatment responses.

## Strengths and limitations

The strength of this study is the comprehensive data from the Danish healthcare registers and the DEGC database which enabled a detailed analysis of survival among Danish gastric cancer patients. The substantial sample size of 2,156 patients and the extended follow-up period facilitated a precise characterization of survival variations across stages and treatment intentions.

However, as a retrospective cohort study, this research has inherent limitations, including incomplete data collection, potential inaccuracies in historical medical records, and the inability to account for changes in treatment protocols over time. A limitation of our study is the variable follow-up time caused by the inclusion of patients up to the end of 2021, with follow-up censored in September 2023. This affects the precision of survival estimates, particularly for patients with more favorable prognosis, where median survival was not reached. While survival patterns are consistent with existing literature, a future repeat analysis would provide more mature survival data and potentially more accurate estimates for these subgroups.

Selection bias is another concern, as treatment allocation was likely influenced by clinical factors not fully captured in the registry, such as performance status, comorbidities, patient preference, surgical candidacy, and occult metastases not recorded in staging. While we adjusted for TNM stage, ASA score, and CCI in multivariate analyses, residual confounding from unmeasured variables cannot be excluded. One example is the observed positive prognostic impact of older age in palliative patients over 80. This likely reflects the selection of elderly patients undergoing diagnostic evaluation rather than a true survival benefit. Similarly, the improved survival of palliative patients with diffuse-type cancer in Stage IVa-b compared with Stage I–III may be explained by comorbidities. Patients with early-stage diffuse cancer may have had significant comorbidities, leading to the choice of palliative care over surgery, ultimately resulting in lower survival rates.

When staging patients, some were grouped due to missing data or registration errors. Additionally, stratification leading to fewer than five patients necessitated further grouping, limiting the feasibility of statistical analysis. While these adjustments may have influenced survival estimates, no significant differences were observed among the grouped stages. These modifications primarily affected surgical patients. In cases where clinical TNM values were unavailable, pathological TNM values were used as a proxy, which did not account for the potential effects of neoadjuvant treatment. Furthermore, statistical power was limited in patients with diffuse cancer due to the smaller sample size, leading to wider confidence intervals and greater uncertainty in the estimates. Lastly, during the observation period, the UICC staging manual transitioned from the 7th to the 8th edition. Since all patients were re-staged according to the 8th edition, this change is not expected to significantly impact overall survival estimates.

## Conclusion

This study highlights the significant impact of treatment strategy, and tumor stage on gastric cancer survival in Denmark. Surgical treatment, particularly with perioperative CT, improved outcomes, while palliative CT or radiotherapy provided survival benefits over best supportive care alone. Adjusted analyses revealed no survival difference between intestinal-type and diffuse-type tumors. These findings emphasize the need for tailored, multimodal treatment approaches to optimize survival and guide clinical decision-making.

## Supplementary Information

Below is the link to the electronic supplementary material.

Supplementary file1 (DOCX 16 KB)

## Data Availability

The data is not available upon request.

## Abbreviations

*ASA*: American Society of Anesthesiologists.
SCT: Surgery ± Chemotherapy.
CT: Chemotherapy.
mOS: Median overall survival
EOX: Epirubicin, oxaliplatin, and capecitabine.
ECX: Epirubicin, cisplatin, and capecitabine.
ECF: Epirubicin, cisplatin, and 5-fluorouracil.
FLOT: 5-Fluorouracil, leucovorin, oxaliplatin, and docetaxel

## Declarations

### Previous communication

The paper is not based on any previous communication to a society or meeting.
