# Supplementary material for: Stage-dependent survival in gastric cancer: a Danish nationwide cohort study
Source: Langenbecks Arch Surg. 2025 Oct 23;410(1):308. doi: 10.1007/s00423-025-03861-y (PMC12546399; doi:10.1007/s00423-025-03861-y)
Supplement: Supplementary file 1 — Supplementary file1 (DOCX 16 KB) [file 423_2025_3861_MOESM1_ESM.docx]

| **Supplementary table 1. Histopathological subtypes** | | | |
| --- | --- | --- | --- |
| **Histology** | | | **n** |
| **Intestinal type** | | |  |
|  | Adenocarcinoma | | 1305 |
|  | Adenocarcinoma, superficially growing | | 10 |
|  | Poorly differentiated adenocarcinoma | | 385 |
|  | Poorly differentiated adenocarcinoma, uncertain if primary or metastatic | | 7 |
|  | Papillary adenocarcinoma | | 2 |
|  |  | |  |
| **Diffuse type** | | |  |
|  | Signet ring cell carcinoma | | 217 |
|  | Poorly cohesive carcinoma | | 27 |
|  |  | |  |
| **Mucinous adenocarcinoma** | | | 24 |
|  |  | |  |
| **Others** | | |  |
|  | Malignant tumor | | 4 |
|  | Malignant tumor cells | | 2 |
|  | Carcinoma | | 86 |
|  | Undifferentiated carcinoma with osteoclast-like giant cells | | 1 |
|  | Squamous cell carcinoma | | 8 |
|  | Adenosquamous carcinoma | | 1 |
|  | |  |  |
| **Missing** | |  | 76 |
|  | |  |  |
| **Total** | |  | 2156 |
| Supplementary table 1.  *illustrates the histopathological distribution of gastric cancers included in this study. The pathological diagnosis is based on the initial biopsy of the tumor.* | | | |
